# Drug Use before and during Pregnancy in Japan: The Japan Environment and Children’s Study

**DOI:** 10.3390/pharmacy5020021

**Published:** 2017-04-10

**Authors:** Hidekazu Nishigori, Taku Obara, Toshie Nishigori, Hirohito Metoki, Mami Ishikuro, Satoshi Mizuno, Kasumi Sakurai, Nozomi Tatsuta, Ichiko Nishijima, Ikuma Fujiwara, Takahiro Arima, Kunihiko Nakai, Nariyasu Mano, Shinichi Kuriyama, Nobuo Yaegashi, Japan Environment & Children’s Study Group

**Affiliations:** 1 Department of Obstetrics and Gynecology, Tohoku University Graduate School of Medicine, Miyagi 9808574, Japan; nishigori@med.tohoku.ac.jp (H.N.); toshienishigori@yahoo.co.jp (T.N.); yaegashi@med.tohoku.ac.jp (N.Y.); 2 Department of Pharmaceutical Sciences, Tohoku University Hospital, Miyagi 9808574, Japan; mano@hosp.tohoku.ac.jp; 3 Environment and Genome Research Center, Tohoku University Graduate School of Medicine, Miyagi 9808575, Japan; hmetoki@med.tohoku.ac.jp (H.M.); m_ishikuro@med.tohoku.ac.jp (M.I.); samizuno@med.tohoku.ac.jp (S.M.); kasakurai69@gmail.com (K.S.); nozomi@med.tohoku.ac.jp (N.T.); nishijii@med.tohoku.ac.jp (I.N.); ifujiwara-endo@umin.ac.jp (I.F.); tarima@med.tohoku.ac.jp (T.A.); nakaik@med.tohoku.ac.jp (K.N.); kuriyama@med.tohoku.ac.jp (S.K.); 4 Tohoku Medical Megabank Organization, Tohoku University, Miyagi 9808575, Japan; 5 Department of Public Health and Hygiene, Tohoku Medical and Pharmaceutical University School of Medicine, Miyagi 9818558, Japan; 6 International Research Institute of Disaster Science, Tohoku University, Miyagi 9808575, Japan

**Keywords:** pregnancy, medication use, supplement use

## Abstract

**Purpose:** To elucidate drug use before and during pregnancy in Japan. **Methods:** The Japan Environment and Children’s Study (JECS) is an ongoing nationwide birth cohort study. We analyzed data from JECS involving cases where drugs were used for 12 months before pregnancy was diagnosed, between the time of diagnosis of pregnancy until week 12 of pregnancy, and after week 12 of pregnancy. **Results:** We analyzed data from 97,464 pregnant women. The percentages of pregnant women who had taken one or more drugs and supplements before diagnosis of pregnancy, between the time of diagnosis of pregnancy until week 12 of pregnancy, and after week 12 of pregnancy, were 78.4%, 57.1%, and 68.8% respectively. Excluding iron supplements, folic acid, and other vitamins and minerals, the percentages of women taking supplements were 75.3%, 36.0%, and 51.7% at each respective time point. The following drugs and supplements were frequently used for 12 months before pregnancy diagnosis: Commercially available antipyretics, analgesics, and/or medicine for treating common cold (34.7%), antipyretics, analgesics, and/or medicine for treating common colds, which were prescribed in hospitals (29.8%), antimicrobial drugs (14.0%), and anti-allergy drugs (12.5%). The following drugs and supplements were frequently used from the time of pregnancy diagnosis until week 12 of pregnancy, and after week 12 of pregnancy: folic acid (28.9% and 26.2%), antipyretics, analgesics and/or medicines for treating common cold, that were prescribed in hospitals (7.8% and 13.3%), Chinese herbal medicines (6.0% and 9.4%, and uterine relaxants (5.1% and 15.2%). **Conclusions:** The analysis of a nationwide cohort study showed that a high percentage of Japanese pregnant women were taking medicinal drugs. Further research is required to elucidate the relationship between drug use during pregnancy and birth defects in Japan.

## 1. Introduction

A number of drugs are known to exert latent harm on pregnant women and their fetuses [1]. According to previous studies on drug use in pregnant women, 40%–90% of these women take at least one drug [2,3,4,5,6,7,8,9,10,11,12,13,14,15,16,17,18,19,20,21,22,23,24]. A study found that the use of a single drug acting on the central nervous system was not related to congenital abnormalities; however, the use of multiple such drugs presented a higher risk of congenital abnormalities [25]. Therefore, it is important for surveys to be conducted on multidrug use and drug use in pregnant women. 

In the Japanese health system, patients mainly obtain their prescribed drugs from community-based pharmacies, based on a prescription made by a doctor in a community clinic or hospital. Patients can also buy over-the-counter drugs in most community pharmacies. To the best of our knowledge, there has been no study based on a large-scale birth cohort survey in Japan. Therefore, there is little information about drug use and safety during pregnancy in Japan, and data from western countries have had to be used. 

To elucidate the status of drug use by pregnant women, we analyzed a dataset from the Japan Environment and Children’s Study, which focused on drug use before and during pregnancy in approximately 100,000 Japanese women.

## 2. Methods 

### 2.1. Study Settings and Subjects

In January 2011, the Japanese Ministry of Environment launched a large-scale cohort epidemiological research project entitled the Japan Environment and Children’s Study (JECS) [26]. JECS is an ongoing nationwide birth cohort study. The plan was to recruit approximately 100,000 pregnant women and their partners over a period of 3 years (i.e., 33,333 per year, which is around 3% of Japanese newborns), collect biological samples, and conduct follow-ups for their children until they reach 13 years of age. The eligibility criteria for participants (expecting mothers) were as follows: (1) They should reside in the study areas at the time of the recruitment, and would be expected to reside continually in Japan for the foreseeable future, (2) expected delivery date should be between 1 August 2011 and mid-2014, and (3) they should be capable to participate in the study without difficulty, i.e., they must be able to comprehend the Japanese language and complete the self-administered questionnaire. Those residing outside the study areas, even if they visited the cooperating health care providers within the study areas, were excluded from the study.

**Figure 1:**
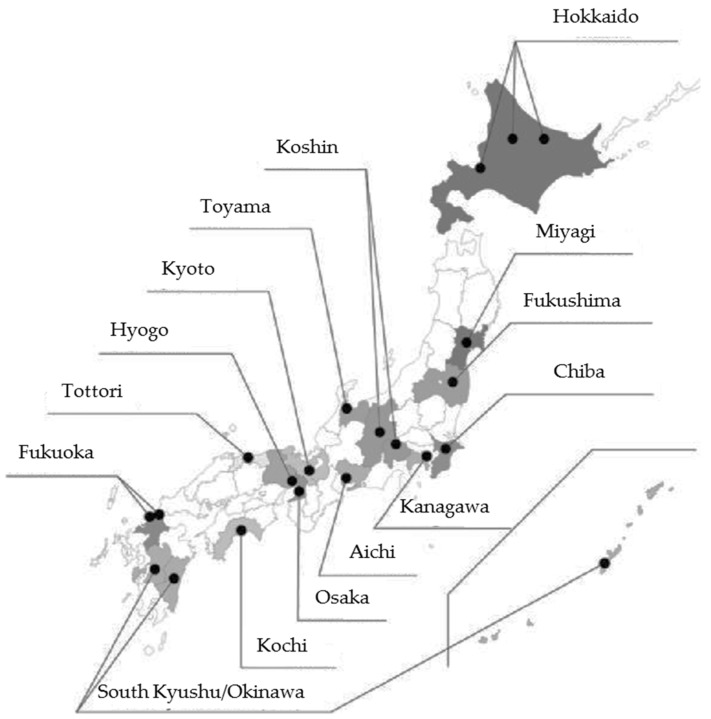
Location of the 15 Regional Centers of the Japan Environment and Children's Study (JECS). The participants were recruited through the 15 regional centers covered by the JECS.

The JECS protocol has been published elsewhere [26], and was approved by the Ministry of Environment’s Institutional Review Board on Epidemiological Studies on 6 April 2010 (no. 100406001), as well as by the Ethics Committees for all participating institutions. Written informed consent was obtained from all participating women and their families. For the JECS, participants were recruited through the 15 Regional Centers located in Hokkaido, Miyagi, Fukushima, Chiba, Kanagawa, Koshin, Toyama, Aichi, Kyoto, Osaka, Hyogo, Tottori, Kochi, Fukuoka, and South Kyushu and Okinawa (Figure 1). Registration for JECS was open between January 2011 and March 2014 for pregnant women on a nationwide basis, and the required data were recorded. In total, about 100,000 pregnant women were enrolled between January 2011 and March 2014. 

The expected date of delivery was determined by comparing the expected date calculated from the crown rump length (CRL) at 8–11 pregnancy weeks, and the one calculated from the last menstrual period. Also, the expected date was calculated from biparietal diameter (BPD) for participants after the correction period with the CRL (around 8–11 weeks) or whose CRL >4 mm. In the case where there was a gap of seven or more days between the expected date of delivery calculated from the start of the last menstrual period and the one calculated from the CRL, the one calculated from the CRL value was adopted. In the case where there was a gap of 10 or more days between the expected dates of delivery as calculated from the start of the last menstrual period and the date calculated from the BPD, the one calculated from the BPD value was adopted.

**Figure 2:**
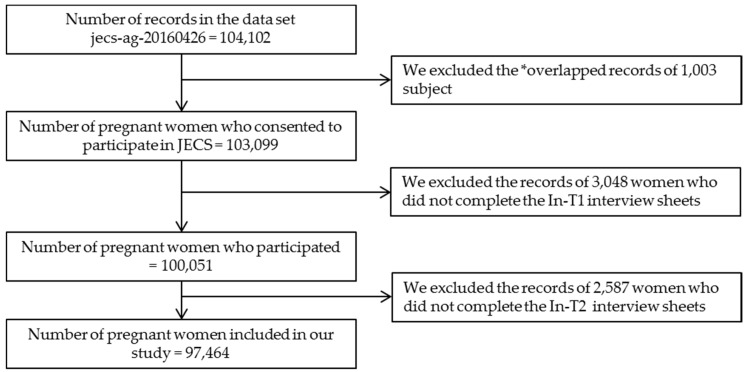
The study analyzed 97,464 pregnant women and provided the raw data for the JECS. * Overlapped records: In the case of multiple pregnancy, the records beyond the first child were excluded.

The present study was based on the data set jecs-ag-20160426, which was released in June 2016. We analyzed this primary dataset from 104,102 records (Figure 2). The data set was created using children as the base. For example, in the case of twin pregnancies, two records (one each for the first and second child) were created for each pregnant woman. Because this study investigated pregnant women, in the case of multiple pregnancies, the records of children beyond the first child were excluded. We analyzed the data from pregnant women who had participated in both “In-T1” and “In-T2” interviews. Of 104,102 records in the jecs-ag-20160426 dataset, the records from 97,464 were analyzed.

### 2.2. Data Collection

All data on drug use were obtained based on two interviews: The “In-T1” interview was performed upon enrollment for mothers during the maternal first trimester or the second trimester (see Appendix A), and the “In-T2” interview was performed during the second or third trimester (see Appendix B). With respect to responses to interviews regarding drug use: During “In-T1” and “In-T2”, a survey was performed by research coordinators through a face-to-face interview for pregnant women, and information was collected from a set medication list (see Appendix C for the full list).

Drug use information was collected during each of the following time periods: (1) Before pregnancy diagnosis (drug use during the 12 months before pregnancy diagnosis), (2) Before week 12 of pregnancy (drug use from pregnancy diagnosis until Week 12 of pregnancy), and (3) After week 12 of pregnancy (drug use from week 12 of pregnancy until the time of the “In-T1” or “In-T2” interview). At period (1), drug use information was collected at the “In-T1” interview. In periods (2) and (3), drug use information was collected at both the “In-T1” and “In-T2” interviews. If a response was recorded at either In-T1 or In-T2, drug use was defined as present.

The gestation week of the In-T1 and In-T2 interviews were calculated back from the date of childbirth, the date of “In-T1” and “In-T2” interview, and the gestation week of birth based on the data set jecs-ag-20160426. Because only the year and month were provided for the dates of childbirth and the dates of the “In-T1” and “In-T2” interviews, the day was defined as the first of the month in all cases.

All data for the study were obtained from the following questionnaires: The “M-T1” questionnaire was performed upon enrollment for mothers during the maternal first or second trimester (M-T1), the“M-T2” questionnaire was performed for mothers during the second or third trimester (M-T2), the “Dr-T1” medical chart review was carried out by a doctor upon enrollment (Dr-T1), and the “Dr-0m” medical chart review was carried out by a doctor immediately after delivery (Dr-0m).

### 2.3. Data Analysis

Based on the M-T1, Dr-T1, M-T2, and Dr-0m questionnaires, the frequency and proportion of pregnant females were calculated, with a focus on the following items: gestation (weeks) of M-T1, gestation (weeks) of M-T2, age, marital status, education, family income, body mass index, smoking, alcohol consumption, parity, fertility treatment, history of spontaneous abortion, history of mental health disorders, hypertension, diabetes, mental health disorders, other pregnancy complications, hypertensive disorders of pregnancy, gestational diabetes, and other obstetric labor complications.

We analyzed the percentages of pregnant women who had taken one or more drugs, excluding supplements such as those for iron, folic acid, and other vitamins and minerals.

All analyses were performed using SAS statistical software, Version 9.4 (SAS Institute Inc., Cary, NC, USA).

## 3. Results

The participants of the 15 Regional Centers of JECS are shown in Table 1. The participants of JECS were distributed nationwide from the north, Hokkaido, to the south, Okinawa. The total number of interviewees in the JECS survey was around 130,000 pregnant women between January 2011 and March 2014, and the number of agreements was 103,099 pregnant women, so the agreement ratio was around 79%. Agreement ratios for each Regional Center have not been determined at this time. In the present analysis, the study population consisted of 104,102 pregnant women. A total of 6638 pregnant women were excluded because of overlapped records for multiple pregnancies, and incomplete “In-T1” and “In-T2” interviews. Thus, we studied 97,464 pregnant women (Figure 2). Characteristics of subjects are shown in Table 2. The mean age and median gestation time of In-T1 and In-T2 in the subjects was 30.8 years, 15.7 weeks, and 27.6 weeks respectively. The median gestation time of M-T1 and M-T2 in the subjects was 15.4 weeks, and 27.7 weeks respectively.

The drugs most commonly used by the women are presented in Table 3. The following drugs and supplements (five upstream items) were frequently used during the 12 months before pregnancy diagnosis: (1) commercially available antipyretics, analgesics, and/or medicine for treating common colds (34.68%), (2) antipyretics, analgesics, and/or medicine for treating common cold prescribed in hospitals (29.82%), (3) antimicrobial drugs (14.02%), (4) anti-allergy drugs (12.53%), and (5) Chinese herbal medicines (8.61%).

The following drugs and supplements (five upstream items) were frequently used from the time of pregnancy diagnosis until week 12 of pregnancy: (1) folic acid (28.89%), (2) antipyretics, analgesics, and/or medicine for treating common cold prescribed in hospitals (7.76%), (3) Chinese herbal medicines (6.02%), (4) uterine relaxants (5.11%), and (5) minerals (4.92%).

The following drugs and supplements (five upstream items) were frequently used from week 12 of pregnancy until the time of the “In-T2” interview: (1) folic acid (26.16%), (2) uterine relaxants (15.21%), (3) antipyretics, analgesics, and/or medicine for treating common cold prescribed in hospitals (13.32%), (4) iron preparations (12.04%), and (5) Chinese herbal medicines (9.41%).

Drug combinations are shown in Table 4. The percentages of pregnant women that took one or more drugs before diagnosis of pregnancy, between the time of diagnosis of pregnancy until week 12 of pregnancy, and after week 12 of pregnancy, were 78.41%, 57.07%, and 68.84%, respectively. A maximum of 10 drugs were combined for the before-pregnancy diagnosis, 11 from the time period between pregnancy diagnosis until week 12 of pregnancy, and 14 for the time period after week 12 of pregnancy.

We analyzed the data, excluding data pertaining to iron supplements, folic acid, and other vitamins and minerals (Table 5). The percentages of pregnant women who had used one or more drugs before diagnosis of pregnancy, from pregnancy diagnosis until week 12 of pregnancy, and after week 12 of pregnancy, were 75.33%, 36.02%, and 51.72%, respectively. A maximum of 10 drugs were combined for the before-pregnancy diagnosis, 10 for the time period between pregnancy diagnosis until Week 12 of pregnancy, and 12 for the time period after Week 12 of pregnancy.

## 4. Discussion

To the best of our knowledge, this study is the first large-scale nationwide investigation on drug use during pregnancy in Japan. Of 101,490 records in the data set, we analyzed the records of 97,464. 

The percentage of pregnant women who had used one or more drugs from the time of pregnancy diagnosis until week 12 of pregnancy was 57.07%. This finding was lower than the percentages reported in studies on pregnant women who had used drugs during the first trimester in Germany (69.7%) [9] and China (75.9%) [22], and higher than the figures reported in studies on pregnant women who had used drugs during the first trimester in Italy (30.7%, 41.0%) [10,11], The Netherlands (43.6%) [12], and Norway (32.8%) [14]. When we excluded iron, folic acid, and other vitamin and mineral supplements from our analysis, the percentage of drug usage from the time of pregnancy diagnosis until Week 12 of pregnancy was lowered to 36.02%. The prevalence of pregnant women who had used drugs during the first trimester in this cohort was similar to that which was reported in studies conducted in the United States (39.0%) [15], lower than that reported in studies conducted in Germany (53.6%) [9] and China (47.7%) [22], and higher than that which was reported in those conducted in Denmark (21.6%) [4]. In comparison to the findings of studies conducted in other countries, the percentage of pregnant women who had used drugs during the early pregnancy period in our cohort was moderate.

The following drugs and supplements (three upstream items) were frequently used from the time of diagnosis of pregnancy until week 12 of pregnancy: (1) folic acid (28.89%), (2) antipyretics, analgesics, and/or medicine for treating common cold prescribed in hospitals (7.76%), and (3) Chinese herbal medicines (6.02%). The following drugs and supplements (three upstream items) were frequently used from week 12 of pregnancy until the time of the “In-T2” interview (the median gestation was 27.6 weeks): (1) folic acid (26.16%), (2) uterine relaxants (15.21%), (3) antipyretics, analgesics, and/or medicines for treating the common cold as prescribed in hospitals (13.32%).

During the first trimester, the most common drugs dispensed in other countries were as follows. Germany: alimentary tract and metabolism, 45% [9]; Italy: hematological, 15.2% [10]; The Netherlands: folic acid, 8.4% [13] and folic acid and derivatives, 6.1% [12]; Norway: antibacterials for systemic use, 9.5% [14]; Denmark: penicillin, 16% [6]; the United States: anti-infectives, 18.3% [15]; China: folic acid, 65% [22].

During the second trimester, the most common drugs dispensed in other countries were as follows. Germany: alimentary tract and metabolism, 46% [9]; Italy: hematological, 30.8% [10]; The Netherlands: iron preparations, 30.6% [13] and 21.0% [12]; Norway: antibacterials for systemic use, 12.0% [14]; Denmark: penicillin, 18% [6]; the United States: anti-infectives, 17.2% [15].

Regarding folic acid ingestion, 6.99% of the pregnant women in our study had consumed folic acid before diagnosis of pregnancy. From the time of diagnosis of pregnancy until week 12 of pregnancy, and from week 12 of pregnancy until the time of the “In-T2” interview, the percentages were 28.89% and 26.16%, respectively. These values before and after pregnancy diagnosis were markedly lower than those reported in the studies conducted in other countries [27]. This indicates that Japanese women of childbearing age should be informed about the need for folic acid intake before pregnancy, to prevent neural tube defects.

The percentage of pregnant women who had received iron preparations from the time of diagnosis of pregnancy until week 12 of pregnancy was 1.71%; this increased after week 12 to 12.04%. This trend is similar but lower than the results reported in studies conducted in Germany (10%, 30%, 40%) [9] and The Netherlands (7.7%, 30.6%, 44.6%) [13], and 5.2%, 21.0%, and 31.5% [12], in a cohort of women who had used drugs during the first, second, and third trimesters respectively. We presumed that the percentage of pregnant women taking iron supplements in Japan was lower than that in Germany and The Netherlands because the frequency of anemia in Japanese pregnant women is low, and treatment guidelines for the use of iron supplements vary. Further research is probably required.

The percentages of pregnant women who had taken Chinese herbal medicines during the time period from the time of diagnosis of pregnancy until week 12 of pregnancy, and after week 12 of pregnancy, were 6.02% and 9.41%; the percentages were high after diagnosis of pregnancy. However, in this survey, the term “Chinese herbal medicines” included all types of preparations, and thus the findings must be carefully interpreted. However, these percentages were similar to the level of use in China during the first trimester (10.1%) [22]. Chinese herbal medicine contains a large number of ingredients, and there is limited information concerning its safety during pregnancy. Safety information must be made available in the future concerning the use of these preparations during pregnancy.

The percentage of pregnant women who had received uterine relaxants during the time period from the time of diagnosis of pregnancy until week 12 of pregnancy was 5.11%; this increased after week 12 of pregnancy to 15.21%. This trend was similar to that reported in the studies conducted in Italy on tocolytic drug use during the first (5.5%), second (10.4%), and third (10.9%) trimesters [10].

The percentages of pregnant women who had used one or more drugs, excluding those pertaining to iron supplements, folic acid, and other vitamins and minerals, during the time period between the time of diagnosis of pregnancy until week 12 of pregnancy, was 36.02%. A maximum of 10 drugs were combined for the time period between diagnosis of pregnancy until week 12 of pregnancy. The percentage of pregnant women who had used four or more drugs between the time period from the time of diagnosis of pregnancy until week 12 of pregnancy was 2.35%. This finding was similar to that reported in the National Birth Defects Prevention Study (2.2%), and lower than that reported in the Slone Epidemiology Center Birth Defects Study (7.5%) conducted in the United States [24].

## 5. Limitations

The subjects of this survey were limited to only pregnant women that participated in JECS voluntarily. Thus, a number of cooperative and health conscious pregnant women likely participated in the survey. Therefore, there is some selection bias here. Furthermore, the self-reporting setup may have resulted in some cases in which drug use was not reported. No validation studies were carried out to assess the accuracy of the reports of medications. Therefore, these findings must be carefully interpreted. 

The “exposure time-period” from after week 12 until the “In-T2” interview was not the same for each participant, as seen from the median (IQR) on gestation of In-T2 in Table 2, and hence, drug use data after week 12 of pregnancy may not have been complete, since some participants may have been interviewed late in the second trimester, or early in the third trimester, and thus, their drug use in the third trimester would not have been collected, or may have been underestimated. Therefore, in general, data on drug use after Week 12 of pregnancy was likely to have been underestimated in this study. 

Some drug types in the dataset were not subclassified (Appendix C). ATC code-based drug and risk classification was not conducted, unlike the surveys conducted in Europe and the United States; it may be difficult to compare our results with those of other surveys because data sources and collection methods differed. 

We could not distinguish between prescription drug use and over-the-counter drug use, because our questionnaire did not completely distinguish between prescription drug use and over-the-counter drug use. 

There has been no prior drug-related study based on a large-scale birth cohort survey in Japan, and these data are a valuable starting point for pregnancy management in Japan.

## 6. Conclusions

The analysis of a large nationwide cohort study showed that a high percentage of Japanese pregnant women were taking medicinal drugs, even when iron, folic acid, and other supplements were excluded. Further research is required to elucidate the relationship between drug use during pregnancy and birth defects in Japan.

## Figures and Tables

**Table 1:** Participants of the 15 Regional Centers and Core Center of the JECS.

Unit Center	*n*	%
Hokkaido	7709	7.9
Miyagi	8775	9.0
Fukushima	12,820	13.2
Chiba	5461	5.6
Kanagawa	6288	6.5
Koshin	7020	7.2
Toyama	5326	5.5
Aichi	5451	5.6
Kyoto	3082	3.2
Osaka	7735	7.9
Hyogo	4951	5.1
Tottori	3007	3.1
Kochi	6817	7.0
Fukuoka	7403	7.6
South Kyusyu and Okinawa	5619	5.8

**Table 2:** Characteristics of study subjects (*n* = 97,464).

		*n*	%
Gestation (weeks) of In-T1	Median(IQR)	15.7 (12.3–22.1)	
	No response	530	0.5
Gestation (weeks) of In-T2	Median(IQR)	27.6 (25.3–31.7)	
	No response	526	0.5
Gestation (weeks) of M-T1	Mean +/− SD	16.8 +/− 7.4	
	Median(IQR)	15.4 (12.4–19.4)	
	No response	859	0.9
Gestation (weeks) of M-T2	Mean +/− SD	28.3 +/− 6.1	
	Median(IQR)	27.7 (25.3–30.4)	
	No response	913	0.9
Age	Mean +/− SD	30.8 +/− 5.0	
	<30 years	39,460	40.5
	30–34 years	33,918	34.8
	≥35 years	23,683	24.3
	No response	403	0.4
Marital status	Married	92,478	94.9
	Single/divorced/widowed	4183	4.3
	No response	803	0.8
Education	Junior high/high school	35,109	36.0
	Technical/junior college	40,517	41.6
	University/graduate school	20,816	21.4
	No response	1022	1.1
Family income	<4,000,000 yen	36,280	37.2
	≥4,000,000 and <6,000,000 yen	29,788	30.6
	≥6,000,000 yen	24,025	24.7
	No response	7371	7.6
Body mass index	<18.5 kg/m^2^	15,595	16.0
	≥18.5 and <25 kg/m^2^	70,483	72.3
	≥25 kg/m^2^	10,301	10.6
	No response	1085	1.1
Smoking	Nonsmokers	56,115	57.6
	Ever smokers	35,632	36.6
	Current smokers	4615	4.7
	No response	1102	1.1
Alcohol consumption	Nondrinkers	33,461	34.3
	Ever drinkers	53,612	55.0
	Current drinkers	9570	9.8
	No response	821	0.8
Parity	Primipara	38,397	39.4
	Multipara	56,786	58.3
	No response	2281	2.3
Fertility treatment	Yes	6463	6.6
	No	90,595	93.0
	No response	406	0.4
History of spontaneous abortion	Yes	18,410	18.9
	No	77,476	79.5
	No response	1578	1.6
History of a mental health disorder	No	89,531	91.9
	Yes	7551	7.8
	No response	382	0.4
Hypertension	No	95,766	98.3
	Yes	1189	1.2
	No response	509	0.5
Diabetes	No	95,909	98.4
	Yes	1046	1.1
	No response	509	0.5
Mental health disorder	No	96,183	98.7
	Yes	772	0.8
	No response	509	0.5
Other pregnancy complication	No	84,305	86.5
	Yes	12,650	13.0
	No response	509	0.5
Hypertensive disorders of pregnancy	No	93,896	96.3
	Mild	2114	2.2
	Severe	945	1.0
	No response	509	0.5
Gestational diabetes	No	94,302	96.8
	Yes	2653	2.7
	No response	509	0.5
Other obstetric labor complication	No	54,648	56.1
	Yes	42,307	43.4
	No response	509	0.5

In-T1: Interview within the maternal first trimester; In-T2: Interview within the second or third trimester; M-T1: Questionnaire within the maternal first trimester; M-T2: Questionnaire within the second or third trimester; IQR: Interquartile range; SD: Standard deviation.

**Table 3:** Drug use before and during pregnancy in Japan from 2011 to 2014 ( *n* =97,464).

		Before Pregnancy Diagnosis		Before Week 12 of Pregnancy		After Week 12 of Pregnancy	
		*n*	%	*n*	%	*n*	%
Antibacterial, Antiviral, Antifungal, Carcinostatic drugs	Antimicrobial	13,668	14.02	3223	3.31	8415	8.63
	Antiviral	3749	3.85	1194	1.23	1521	1.56
	Antifungal	936	0.96	1112	1.14	1904	1.95
	Carcinostatic	628	0.64	503	0.52	65	0.07
Corticosteroids	Corticosteroids: oral administration, inhalation, infusion	3008	3.09	1120	1.15	1660	1.70
	Corticosteroids: external use, enema	5415	5.56	2756	2.83	4283	4.39
Antipyreic, Analgesic drugs	Antipyretic, Analgesic, Medicine for common cold: prescription	29,064	29.82	7498	7.82	12,982	13.32
	Antipyretic, Analgesic, Medicine for common cold: over the counter	33,799	34.68	3035	3.16	1162	1.19
	Poultice which the analgesic is included in	4352	4.47	926	0.97	1711	1.76
Antirheumatic drugs	Immunosuppressant, Immunoregulation	650	0.67	470	0.48	144	0.15
	Infliximab, Etanercept	42	0.04	20	0.02	21	0.02
	Antirheumatic drug unidentified in detail	14	0.01	4	0.00	4	0.00
Antiallergy drugs	Antiallergic drug (oral administration, inhalation, nasal drip, tape, Antihistaminic)	12,213	12.53	2688	2.76	5389	5.53
Respiratory drugs	β stimulative (oral administration, inhalation)	528	0.54	286	0.29	541	0.56
	Nontypable inhalant	144	0.15	51	0.05	77	0.08
	Antitussive, Expectorant	4711	4.83	1362	1.40	4131	4.24
	Theophylline	346	0.36	88	0.09	189	0.19
	Other respiratory drug	207	0.21	89	0.09	181	0.19
Antidiabetic drugs, Antihyperlipidemic drugs	Insulin preparation	100	0.10	163	0.17	289	0.30
	Hypoglycemic tablet	207	0.21	96	0.10	35	0.04
	Antihyperlipidemic	79	0.08	27	0.03	26	0.03
	Antigout	9	0.01	2	0.00	2	0.00
Hormone-related drugs	Thyroid hormone preparation/levothyroxine sodium	642	0.66	640	0.66	710	0.73
	Antithyroid/Thiamazole	360	0.37	309	0.32	311	0.32
	Other hormone drugs	2245	2.30	966	0.99	340	0.35
Blood-related drugs	Iron preparartion	1243	1.28	1670	1.71	11,736	12.04
	Other blood-related	1030	1.06	2181	2.24	2040	2.09
Cardiovasucular drugs	Antihypertensive (including diuretic)	269	0.28	145	0.15	291	0.30
	Pressor	32	0.03	8	0.01	18	0.02
	Antiarrhythmic, Antianginal	51	0.05	49	0.05	68	0.07
	Heart failure therapeutic	3	0.00	2	0.00	3	0.00
	Other caridiovasucular drugs	181	0.19	142	0.15	167	0.17
Gastrointestinal drugs	Antiulcer (Proton pump inhibitor, H2 blocker)	1397	1.43	345	0.35	686	0.70
	General gastrointestinal agents	8107	8.32	2777	2.85	5036	5.17
	Other gastrointestinal agents	2325	2.39	1416	1.45	2176	2.23
Psychoactive drugs	Selective serotonin reuptake inhibitors (SSRI)	518	0.53	173	0.18	149	0.15
	Antidepressant drug except the SSRI	265	0.27	91	0.09	79	0.08
	Antianxiety	992	1.02	326	0.33	372	0.38
	Sleeping pill	946	0.97	228	0.23	238	0.24
	Antipsychotic	233	0.24	104	0.11	119	0.12
	Valproic acid	180	0.18	66	0.07	68	0.07
	Antiepileptic except the above	142	0.15	117	0.12	133	0.14
	Lithium carbonate	30	0.03	3	0.00	4	0.00
	Other psychoactive drugs	116	0.12	32	0.03	33	0.03
Perinatal drugs	Utero relaxants	522	0.54	4982	5.11	14,822	15.21
	Utero-tonic	795	0.82	188	0.19	279	0.29
	Ovulation inducing	3846	3.95	277	0.28	22	0.02
	Other perinatal related drugs	2420	2.48	2672	2.74	1964	2.02
Other drugs	Anesthetic, pain block injection	932	0.96	112	0.11	447	0.46
	Chinese herbal medicines	8389	8.61	5865	6.02	9175	9.41
	External application (non-identified contents)	4905	5.03	1972	2.02	3966	4.07
	Injection, Drip infusion (non-identified contents)	1460	1.50	1043	1.07	942	0.97
	Bone & Calcium metabolism	93	0.10	95	0.10	126	0.13
	Antimigraine headache	308	0.32	60	0.06	87	0.09
	Muscle relaxant	269	0.28	23	0.02	21	0.02
	Antiemetic drug	985	1.01	1802	1.85	1316	1.35
	AntiParkinson	112	0.11	38	0.04	12	0.01
	Hemorrhoids	297	0.30	377	0.39	1076	1.10
Supplements, vitamins/minerals	Folic acid	6810	6.99	28,153	28.89	25,494	26.16
	Vitamin A	122	0.13	70	0.07	72	0.07
	Vitamin B	2648	2.72	2282	2.34	2894	2.97
	Vitamin C	2794	2.87	1536	1.58	1793	1.84
	Vitamin D	131	0.13	111	0.11	148	0.15
	Vitamin E	636	0.65	277	0.28	267	0.27
	Mineralas	1760	1.81	4797	4.92	6751	6.93
	Multi vitamins supplement	3766	3.86	2956	3.03	3059	3.14
	Total supplement	2620	2.69	2680	2.75	3380	3.47
Illegal drugs	Marijuana	2	0.00	1	0.00	3	0.00
	Psychostimulant	1	0.00	0	0.00	2	0.00
	Ecstasy	0	0.00	0	0.00	1	0.00
	Thinner	1	0.00	0	0.00	0	0.00
	Toluene	2	0.00	0	0.00	0	0.00
	Other illegal drugs	2	0.00	1	0.00	0	0.00
Vaccines		1431	1.47	318	0.33	897	0.92
Drugs not included in the list mentioned above		5064	5.20	3011	3.09	5272	5.41
Forgot the drug name		1342	1.38	486	0.50	607	0.62

Before diagnosis of pregnancy: Drug use during the 12 months before diagnosis of pregnancy, Before week 12 of pregnancy: Drug use between the time from diagnosis of pregnancy until week 12 of pregnancy, After week 12 of pregnancy: Drug use from week 12 of pregnancy until the “In-T1” or “In-T2” interview.

**Table 4:** Percentage of pregnant women taking more than one medication including supplements, folic acid, and other vitamins and minerals (*n* = 97,464).

Number of Items	Before Pregnancy Diagnosis	Before Week 12 of Pregnancy	After Week 12 of Pregnancy
	%	%	%
0	21.59	42.93	31.16
1	25.54	27.61	25.51
2	20.06	15.46	18.49
3	19.34	9.75	14.30
4	5.14	2.60	5.16
5	3.32	0.97	2.57
6	2.56	0.43	1.39
7	1.51	0.17	0.69
8	0.69	0.06	0.40
9	0.24	0.02	0.19
10	0.02	0.01	0.08
11	-	0.003	0.03
12	-	-	0.01
13	-	-	0.005
14	-	-	0.002
>1	78.41	57.07	68.84

**Table 5:** Percentage of pregnant women taking more than one medication excluding supplements, folic acid, and other vitamins and minerals (*n* = 97,464).

Number of Items	Before Pregnancy Diagnosis	Before Week 12 of Pregnancy	After Week 12 of Pregnancy
	%	%	%
0	24.67	63.98	48.28
1	28.46	20.54	25.22
2	20.71	9.03	13.65
3	15.39	4.10	7.52
4	4.50	1.39	2.71
5	2.75	0.58	1.32
6	1.80	0.26	0.68
7	1.08	0.08	0.35
8	0.50	0.03	0.17
9	0.14	0.01	0.07
10	0.01	0.005	0.03
11	-	-	0.01
12	-	-	0.003
>1	75.33	36.02	51.72

**Figure A1:**
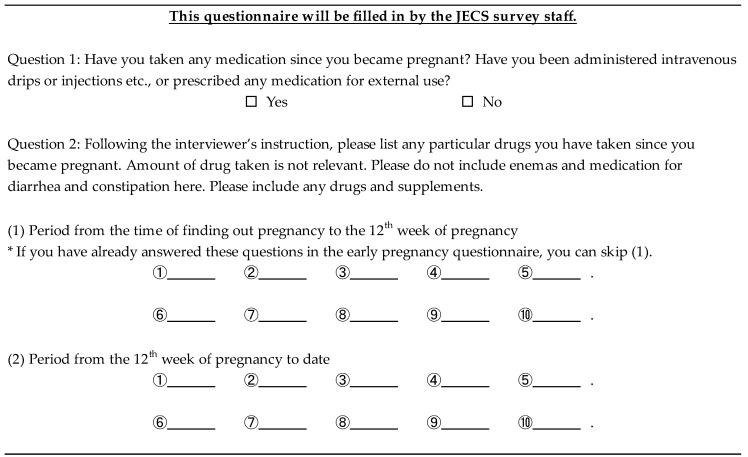
Medication questionnaire (In-T1).

**Figure A2:**
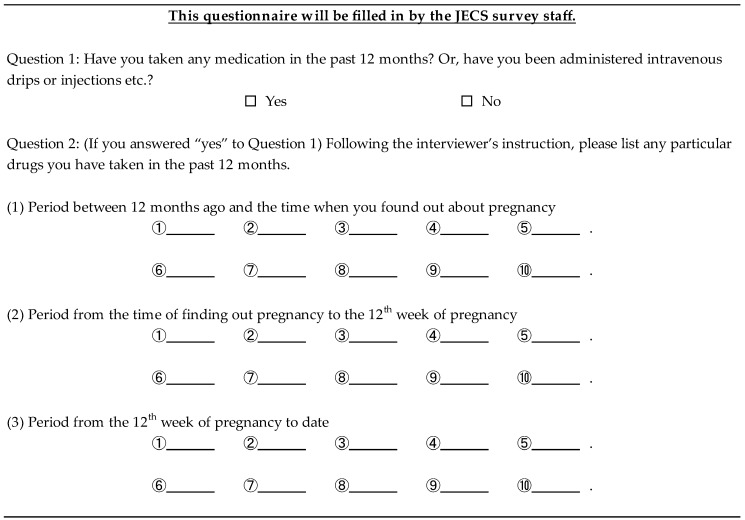
Medication questionnaire (In-T2).

**Figure A3:**
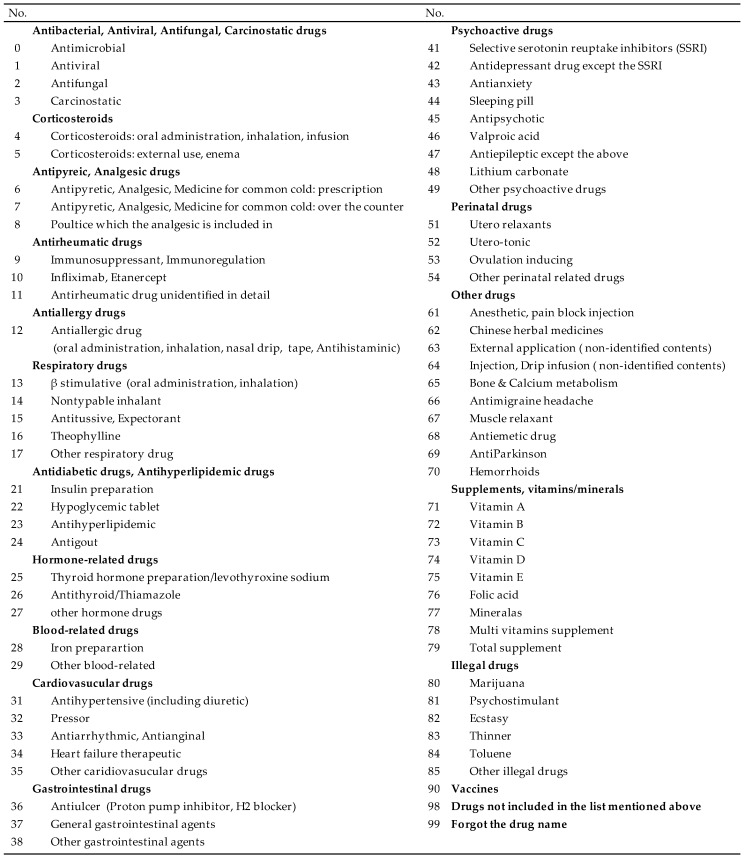
Medication list.
